# Management venöser Aneurysmen und deren gefäßchirurgische Therapiemöglichkeiten: Auswahl repräsentativer Fallkonstellationen mit Erfahrungen eines gefäßchirurgischen Zentrums

**DOI:** 10.1007/s00104-024-02191-x

**Published:** 2024-11-14

**Authors:** U. Barth, M. Stojkova, F. Meyer, Z. Halloul

**Affiliations:** 1 Arbeitsbereich Gefäßchirurgie, Klinik für Allgemein‑, Viszeral- und Gefäßchirurgie, Universitätsklinikum Magdeburg A. ö. R., Leipziger Str. 44, 39120 Magdeburg, Deutschland; 2 Klinik für Radiologie und Nuklearmedizin, Universitätsklinikum Magdeburg A. ö. R., Magdeburg, Deutschland; 3 Klinik für Allgemein‑, Viszeral‑, Gefäß- und Transplantationschirurgie, Universitätsklinikum Magdeburg A. ö. R., Magdeburg, Deutschland

**Keywords:** Venöses Aneurysma, Lungenembolie, Tangentiale Aneurysmektomie, Direkte orale Antikoagulanzien (DOAK), Endophlebohypertrophie, Endophlebosklerose, Venous aneurysm, Pulmonary embolism, Tangential aneurysmectomy, Direct oral anticoagulants (DOAC), Endophlebohypertrophy, Endophlebosclerosis

## Abstract

**Einleitung:**

Venöse Aneurysmen bilden in der Gefäßchirurgie eine seltene Entität, die meist in Einzelfallserien und daraus generierten Metaanalysen beschrieben wird. Die Behandlungskonzepte sind vielfältig, hervorgehoben wird die operative Therapie wegen des Thromboserisikos und des Risikos für Lungenembolien. Uneinigkeit besteht hinsichtlich der postoperativen Notwendigkeit und Dauer der Antikoagulation.

**Methode:**

Serie einer konsekutiven Patientenkohorte mit venösem Aneurysma der letzten 18 Jahre einer Gefäßchirurgie der hochspezialisierten Versorgung mit zentrumsgleichen Strukturen inklusive einer Bewertung der eigenen Erfahrungen aus der täglichen gefäßchirurgischen Praxis im Lichte einer aktuellen Literaturauswahl zu möglichen und vor allem etablierten diagnosespezifischen Therapiekonzepten.

**Ergebnisse:**

Zwischen 2005 und 2023 wurden insgesamt 11 Fälle venöser Aneurysmen bei Patienten im Alter von 30 bis 84 (Mittelwert: 52,5; Median: 50) Jahre(n) eruiert, wobei bei einem Patienten nach 2 Jahren ein Rezidiv operiert werden musste. Das Geschlechterverhältnis betrug 7:3 (m:w). Von der anatomischen Region war die V. poplitea mit 36,4 % am häufigsten betroffen, gefolgt von der V. jugularis interna und V. axillaris/subclavia mit je 18,2 %. Ein Aneurysma der V. cava inferior, der V. iliaca communis und V. cubiti media traten nur einmal auf. In 9 Fällen erfolgte eine operative Versorgung der Aneurysmen. Dabei kamen als Operationsmethoden eine **i)** tangentiale Resektion der Aneurysmawand und fortlaufende Raffungsnaht, **ii)** Resektion des Aneurysmas und Interposition einer 8‑mm-GORE-TEX®Vascular-Graft-Prothese (W.L. Gore, Putzbrunn, Deutschland), **iii)** Ligatur des Aneurysmas und **iv)** Ligatur mit anschließender Resektion des Aneurysmas zur operativ-technischen Anwendung.

**Schlussfolgerung:**

Die Seltenheit des Auftretens der venösen Aneurysmen sollte Anlass geben, diese Fälle zentral zu registrieren und auszuwerten (ggf. bundesweites Register). Die chirurgische Behandlung ist meist unproblematisch und mit wenigen Komplikationen behaftet. Das Risiko von Lungenembolien scheint bei venösen Aneurysmen der Extremitäten, Beckenvenen und V. cava inferior deutlich erhöht zu sein, während venöse Aneurysmen im Kopf- und Halsbereich deutlich weniger dazu neigen. Die peri- und postoperative Antikoagulation hat sich an die Entwicklung der Antikoagulanzien angepasst zugunsten der Therapie mit direkten oralen Antikoagulanzien (DOAKs). Eine unmittelbar postoperative(s) „low-dose“-Heparinisierung und anschließendes therapeutisches Bridging mit einem niedermolekularen Heparin vor Einstellung auf ein ambulantisierfähiges Antikoagulans scheint die perioperative Phase bezüglich operationsbedingter, insbesondere thrombotischer Komplikationen (aber auch Blutung) nach eigener Erfahrung abzusichern.

## Abstract (en)

**Introduction:**

Venous aneurysms are a rare entity in vascular surgery, which are mostly described in individual case series and meta-analyses generated from them. The treatment concepts are diverse and surgical treatment is highlighted due to the risk of thrombosis and pulmonary embolism. There is still an ongoing debate regarding the postoperative necessity and duration of anticoagulation.

**Method:**

Case series of a consecutive patient cohort with venous aneurysms from the last 18 years in a center of (highly specialized care) vascular surgery including i) own experiences obtained in daily vascular surgical practice and ii) a selected and current literature search of relevant references on possible and, in particular, established diagnosis-specific therapeutic concepts.

**Results:**

Between 2005 and 2023, a total of 11 cases of venous aneurysms were reported in patients aged 30–84 years (mean: 52.5, median: 50), with 1 patient requiring surgery for a recurrence after 2 years. The gender ratio was 7:3 (m:f) and the popliteal vein was the most frequently affected anatomical region with 36.4%, followed by the internal jugular vein and axillary/subclavian vein each with 18.2%. Aneurysms of the inferior vena cava, the common iliac vein and the cubital vein occurred only once. Surgical treatment of the aneurysms was performed in 9 cases. The surgical methods used were **i)** tangential resection of the aneurysm wall and continuous purse-string suture, **ii)** resection of the aneurysm and interposition of an 8‑mm GORE-TEX® vascular graft prosthesis (Gore, Putzbrunn, Germany), **iii)** ligation of the aneurysm and **iv)** ligation with subsequent resection of the aneurysm.

**Conclusion:**

The rarity of venous aneurysms should be a reason to register these cases centrally (possibly, nationwide diagnosis-related register). Surgical treatment is usually unproblematic and associated with few complications. The risk of pulmonary embolism appears to be significantly increased in venous aneurysms of the extremities, pelvic veins and inferior vena cava, while venous aneurysms of the head and neck are significantly less prone to this. Perioperative and postoperative anticoagulation has been adapted to the development of specific anticoagulants and novel drugs, in favor of treatment with direct oral anticoagulants (DOAC). In personal experience, immediate postoperative heparin perfusion (low dose) and subsequent therapeutic bridging with low-molecular-weight heparin before switching to an anticoagulant for outpatient clinic-based care appears to safeguard the perioperative phase with respect to keeping the surgery-related complication rate (e.g., thrombosis, bleeding) low.

## Hintergrund

Venöse Aneurysmen bilden eine seltene Entität der vaskulären Erkrankungen, sodass bei der klinischen Therapie ein großer Konsens, jedoch wenig Evidenz vorhanden ist. Metaanalysen aus unterschiedlich gearteten Fallserien sind schwierig zu beurteilen. So kann die Verifizierung der Pathogenese, der Risikofaktoren, der optimalen Therapie und Antikoagulation weiter nur anhand der Erfahrungen der Gefäßchirurgen^1^ abgeleitet werden. Noppeney et al. [14] berichteten über 1199 publizierte Fälle venöser Aneurysmen bis 2016 unterschiedlichster Lokalisation. Am häufigsten waren Aneurysmen der extrahepatischen V. portae (*n* = 247), gefolgt von Aneurysmen der V. poplitea (*n* = 223) und am dritthäufigsten Aneurysmen der V. jugularis interna oder externa (*n* = 143).

Die Pathogenese kann unterschiedlicher Natur sein, so werden eine venöse Hypertension, eine entzündliche oder infektiöse Genese, eine angeborene Venenwandschwäche, ein mechanisches Trauma und andere hämodynamische Veränderungen als Auslöser der degenerativ-dilatativen Veränderungen angeschuldigt [27].

Histologisch weisen Venenaneurysmen eine Fragmentierung der elastischen Lamellen mit Verlust glatter Muskelzellen sowie eine erhöhte Expression von MMP‑2, MMP‑9 und MMP-13 auf [4].

Ziel der Arbeit: Der eigene Umgang mit dieser seltenen Erkrankung in einem tertiären Gefäßzentrum soll anhand der Auswertung der eigenen Fallsammlung diskutiert werden.

## Material und Methoden

Die Fallserie einer konsekutiven Patientenkohorte mit gesichertem venösen Aneurysma über einen definierten Beobachtungszeitraum soll auf Basis aktueller und einschlägiger wissenschaftlicher Referenzen, recherchiert in PubMed®, und eigener operativer und klinischer Erfahrungen

- das entscheidungstechnische Herangehen,
- das taktische Vorgehen,
- die operativen Möglichkeiten (sowie)
- deren perioperatives Management anhand 10 ausgewählter Fallkomplexe diverser
  - Gefäßsegmente bzw. Körperregionen und
  - Patientenkonstellationen (Alter, Zeitgang)

darstellen **i)** zur chirurgischen Qualitätssicherung, **ii)** zur Reflexion des gefäßchirurgischen Alltags und **iii)** als Beitrag zur gefäßchirurgischen Versorgungsforschung.

### Stellungnahme

Die Studie wurde gemäß den Richtlinien der Deklaration von Helsinki für die biomedizinische Forschung aus dem Jahr 1964 und deren weiteren Änderungen, gemäß den Anweisungen der institutionellen Ethikkommission sowie gemäß den Regeln der „Guten Klinischen Praxis und Forschung“ durchgeführt.

Jeder Patient unterzeichnete vor dem chirurgischen Eingriff eine Einverständniserklärung, nachdem der verantwortliche Chirurg den Patienten in einem ausführlichen persönlichen Gespräch über die einzelnen Schritte und möglichen Komplikationen des chirurgischen Eingriffs aufgeklärt und alle Fragen und Bedenken des Patienten verständlich beantwortet hatte.

Die Daten enthalten personenbezogene Informationen gemäß dem Datenschutzgesetz und können aus ethischen Gründen nicht ohne entsprechende Genehmigungen öffentlich zugänglich gemacht werden. Entsprechend § 17 Abs. 1 S. 2 des Krankenhausgesetzes des Landes Sachsen-Anhalt wurden die im Rahmen der Krankenhausbehandlung erhobenen und gespeicherten Patientendaten vor ihrer weiteren Verarbeitung anonymisiert und konnten dadurch auch ohne Einwilligung bei berechtigtem Interesse der Allgemeinheit an der Durchführung des Forschungsvorhabens verwendet werden.

## Ergebnisse (fallserienbasierte Kasuistiken)

### Venöse Aneurysmen der unteren Extremitäten

Die häufigsten gemeldeten Fälle venöser Aneurysmen betreffen die unteren Extremitäten [22]. Die poplitealen venösen Aneurysmen machen dabei den größten Anteil aus [29]. Die Gefährlichkeit eines venösen poplitealen Aneurysmas liegt im 80 %igen Risiko einer Lungenembolie [19]. Da selbst unter Antikoagulation weiter das Risiko rezidivierender Lungenembolien besteht [22], gilt die operative Versorgung als weitestgehend unumstritten. Die Unterscheidung erfolgt in primäre und sekundäre Aneurysmen, wobei die Ätiologie primärer Aneurysmen unklar ist und sekundäre Aneurysmen auf Basis eines venösen Hochdrucks oder als Folge eines direkten Traumas entstehen können [16].

#### Fallbericht 1

**Abb. 1:**
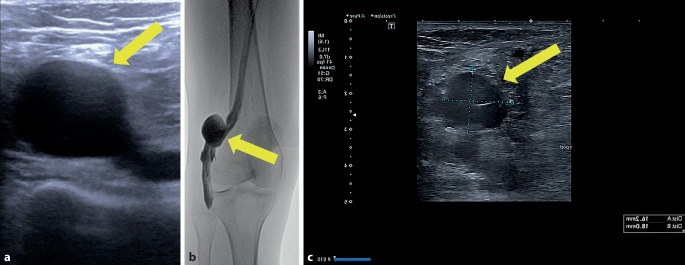
Fall 1: Venöses Aneurysma der V. poplitea rechts. **a** Sonographische Darstellung des Aneurysmas der V. poplitea im B‑Bild (*gelber Pfeil*), **b** Phlebographische Darstellung des venösen Poplitealaneurysmas rechts (*gelber Pfeil*), **c** Postoperative Ultraschallkontrolle des versorgten Aneurysmas der V. poplitea im Querschnitt mit 1,6 cm (*gelber Pfeil*)

Eine 55-jährige Patientin erlitt eine Tibiakopftrümmerfraktur links, die mit einem gelenkübergreifendem AO(Arbeitsgruppe Osteosynthese)-Fixateur versorgt wurde. Daraufhin entwickelte die Patientin eine Unterschenkel(US)-Venenthrombose im Bereich der Vv. fibulares et posteriores links. Nach definitiver Versorgung der Fraktur wurde die Patientin auf Apixaban (Eliquis®, Pfizer, Deutschland) 2 × 5 mg eingestellt. In der Kontrollduplexsonographie nach 4 Monaten zeigte sich neben kleinen Residuen der stattgehabten tiefen Venenthrombose (TVT) des linken US die rechte V. poplitea auf ca. 3 cm aneurysmatisch erweitert (Abb. 1a). Die Antikoagulation wurde fortgeführt. Die erneute Kontrolle nach 4 Monaten ergab einen konstanten Befund eines 2,5 × 2,5 × 3,5 cm messenden Aneurysmas der V. poplitea rechts, woraufhin die gefäßchirurgische Vorstellung erfolgte. Zur Befundverifizierung erfolgte die Durchführung einer Phlebographie (Abb. 1b). Therapeutisch erfolgte die operative tangentielle Resektion und Raffungsnaht auf 3 cm Länge. Nach komplikationslosem postoperativen Verlauf wurde die Patientin erneut auf Apixaban eingestellt. Die histologische Untersuchung des Resektates ergab ein ektatisches Venenresektat mir regulärem Wandaufbau ohne Atypien. Das erste „follow-up“ nach einem halben Jahr im Bereich der resezierten V. poplitea ergab einen sonographisch gemessenen Durchmesser von 1,6 cm ohne thrombotisches Material (Abb. 1c). Es wurde die Fortführung der Antikoagulation für ein weiteres Jahr empfohlen.

#### Fallbericht 2

**Abb. 2:**
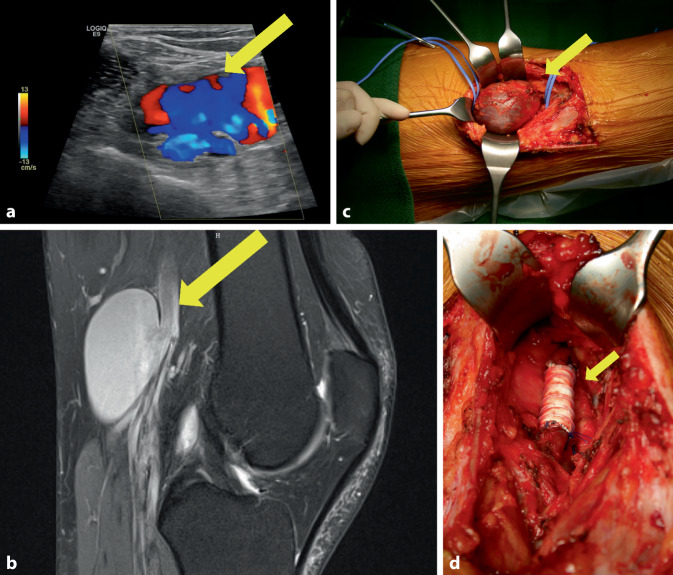
Fall 2: Venöses Poplitealaneurysma links. **a** Darstellung des Aneurysmas der V. poplitea links in der farbkodierten Duplexsonographie (*gelber Pfeil*), **b** Darstellung des venösen Aneurysmas in der MR-Angiographie in sagittaler Schnittrichtung (*gelber Pfeil*), **c** Intraoperative Darstellung des venösen Poplitealaneurysmas während der Primäroperation (*gelber Pfeil*), **d** Intraoperative Darstellung der rekonstruierten V. poplitea mittels GORE-TEX®Vascular Graft-Prothese (W.L. Gore, Putzbrunn, Deutschland) auf einer Länge von ca. 4 cm (*gelber Pfeil*)

Ein 44-jähriger Mann klagte über eine seit mehreren Wochen bestehende und zunehmende Luftnot mit Herzrasen. Bei der Vorstellung in der Notaufnahme fiel eine deutliche Erhöhung des D‑Dimers auf, sodass bei Verdacht auf eine Lungenarterienembolie eine CT-Thorax-Untersuchung erfolgte. Diese konnte Lungenembolien auf Segment- und Subsegmentebene beidseits bestätigen. Echokardiographisch zeigte sich eine deutliche Rechtsherzbelastung. Die sich anschließende Diagnostik mittels farbkodierter Duplexsonographie (Abb. 2a) und MRT ergab ein 3 × 6 cm großes Aneurysma der V. poplitea (Abb. 2b) links. In kurzem zeitlichen Intervall erfolgte die Resektion des venösen Aneurysmas und Raffungsnaht (Abb. 2c). Nach komplikationslosem Verlauf wurde der Patient unter Antikoagulation mit Apixaban und Kompressionstherapie mit einem Kompressionsstrumpf der Klasse II in die ambulante Betreuung entlassen. Es erfolgte die jährliche ambulante Verlaufskontrolle, wobei sich 2 Jahre später in einer duplexsonographischen Verlaufskontrolle eine erneute aneurysmatische Aufweitung der V. poplitea links zeigte. Die sich anschließende Phlebographie bestätigte den Befund, sodass eine erneute operative Therapie mit dem Patienten vereinbart wurde. Es erfolgte die Resektion des venösen Aneurysmas der V. poplitea links und Ersatz mittels 8 mm durchmessender beringter GORE-TEX®Vascular-Graft-Prothese (W.L. Gore, Putzbrunn, Deutschland) auf einer Länge von ca. 4 cm (Abb. 2d). Histologisch zeigten sich mäßig myxoid degenerierende Aneurysmawandanteile mit polarisationsoptischem Fremdmaterial ohne Anhalt für Entzündung, Thrombosierung oder Malignität. Auch hier verlief der postoperative Zeitraum komplikationslos. Die Antikoagulation mit Apixaban wurde fortgeführt. Die jährlichen ambulanten follow-up-Untersuchungsintervalle konnten nach 4 Jahren bei unauffälligem Befund unter reduzierter Apixabandosis gestreckt werden.

### Venöse Aneurysmen im Kopf-Hals-Bereich

Aneurysmen im jugular-venösen Bereich sind ebenfalls sehr selten und können die V. jugularis interna, V. jugularis externa und die vorderen Jugularvenen betreffen, wobei die V. jugularis interna am häufigsten ein Aneurysma aufweist [2]. Klinisch zeigen sie sich am häufigsten als asymptomatische Weichteilmassen, die bei einem Valsalva-Manöver an Größe zunehmen [13]. Die in der Literatur beschriebenen Fälle wurden zum Teil aufgrund der Kosmetik oder der Befürchtung, dass sich die Läsion ausdehnen würde, mit chirurgischer Exzision oder Ausschluss behandelt, während die übrigen Patienten nichtchirurgisch mit bildgebender Nachsorge kontrolliert wurden. Bei keinem Patienten wurde eine Komplikation, einschließlich venöser Thrombembolie (VTE) und Ruptur, festgestellt [22].

#### Fallbericht 3

**Abb. 3:**
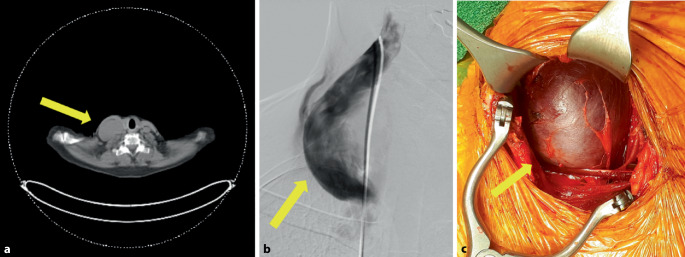
Fall 3: Venöses Aneurysma der V. jugularis interna rechts. **a** Darstellung des Aneurysmas mittels F‑18-Cholin-PET/CT (*gelber Pfeil*), **b** Selektive phlebographische Darstellung des V.-jugularis-Aneurysmas (*gelber Pfeil*), **c** Intraoperativer Befund des Aneurysmas (*gelber Pfeil*)

Im Rahmen der Diagnostik eines primären Hyperparathyreodismus mit Nephrolithiasis und Osteoporose bei Nebenschilddrüsenadenom rechts-kaudal mittels F‑18-Cholin-PET/CT (Positronenemmissionstomographie/Computertomographie; Abb. 3a) und selektiver Halsvenenkatheterisierung (Abb. 3b) zeigte sich bei der 62-jährigen Patientin ein Aneurysma der V. jugularis interna rechts. Nebenbefundlich litt die Patientin weiter an einem Pseudoxanthoma elasticum (Grönblad-Strandberg-Syndrom). Zusammen mit der Resektion des Nebenschilddrüsenadenoms rechts-kaudal erfolgte die tangentiale Resektion des Aneurysmas und Raffungsnaht auf 10 cm Länge (Abb. 3c). Postoperativ entwickelte sich ein Serom im Operationsgebiet bei ansonsten reizlosen Wundverhältnissen, welches konservativ behandelt wurde. Die histologische Aufarbeitung des resezierten Präparates zeigte eine intramurale Texturstörung und luminale Anteile eines Parietalthrombus ohne Infektion oder Malignität [5].

#### Fallbericht 4

**Abb. 4:**
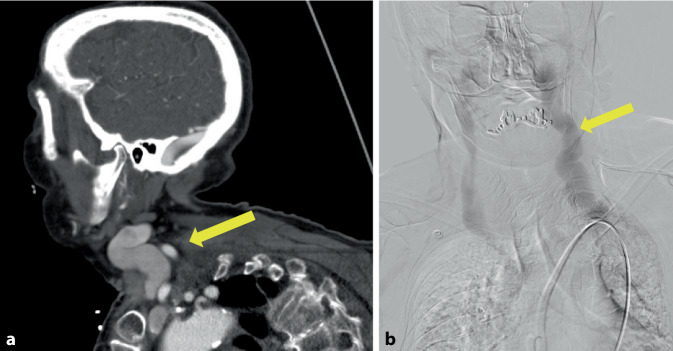
Fall 4: Aneurysma der V. jugularis interna links. **a** CT-Angiographie in der venösen Phase und sagittaler Schichtung mit Darstellung der aneurysmatischen V. jugularis interna links (*gelber Pfeil*), **b** Venöse digitale Subtraktionsangiographie der aneurysmatischen linken V. jugularis interna (*gelber Pfeil*)

Eine 84-jährige Patientin stellte sich notfallmäßig mit einer temporären Sprachstörung in der Notfallambulanz vor. Zudem bemerkte sie anschließend eine Schwellung des Halsbereiches links. Unter dem Verdacht einer Karotisdissektion erfolgte die CT-Angiographie des Halses und Kopfes. Als Ursache für die Schwellung im linken Halsdreieck wurde eine aneurysmatische Aufweitung der V. jugularis interna verifiziert (Abb. 4a). Zum differenzialdiagnostischen Ausschluss einer zervikalen arteriovenösen (AV-)Fistel erfolgte eine digitale Subtraktionsangiographie (DSA) der zervikalen Arterien und Venen, die selbige ausschloss und die aneurysmatische Erweiterung der V. jugularis interna erneut bestätigte (Abb. 4b). In einem ausführlichen Aufklärungsgespräch über Risiko und Nutzen einer operativen Behandlung entschied man sich zusammen mit der Patientin für ein weiteres konservatives Vorgehen.

### Venöse Aneurysmen der oberen Extremitäten

Das Vorhandensein einer nichtpulsierenden Weichteilmasse mit einer offensichtlichen Ausdehnung der oberflächlichen Venen in die obere Extremität sollte als mögliches venöses Aneurysma angesehen und weitere diagnostische Verfahren durchgeführt werden [11]. Die Größe der Aneurysmen nimmt bei der Elevation der Extremität ab und bei Depression an Größe zu. Zu den diagnostischen Maßnahmen, die im Bewertungsprozess verwendet werden sollten, gehören Duplexsonographie, Venographie, MRT-, CT-Scans und gelegentlich eine Arteriographie [13]. Während die operative Versorgung venöser Aneurysmen der unteren Extremitäten weitestgehend unstrittig ist, besteht bei Aneurysmen der oberen Extremitäten Uneinigkeit, ob eine aggressive chirurgische Behandlung gerechtfertigt ist, insbesondere bei asymptomatischen Patienten, da eine Resektion nicht komplett ohne Morbidität verläuft [24].

#### Fallbericht 5

**Abb. 5:**
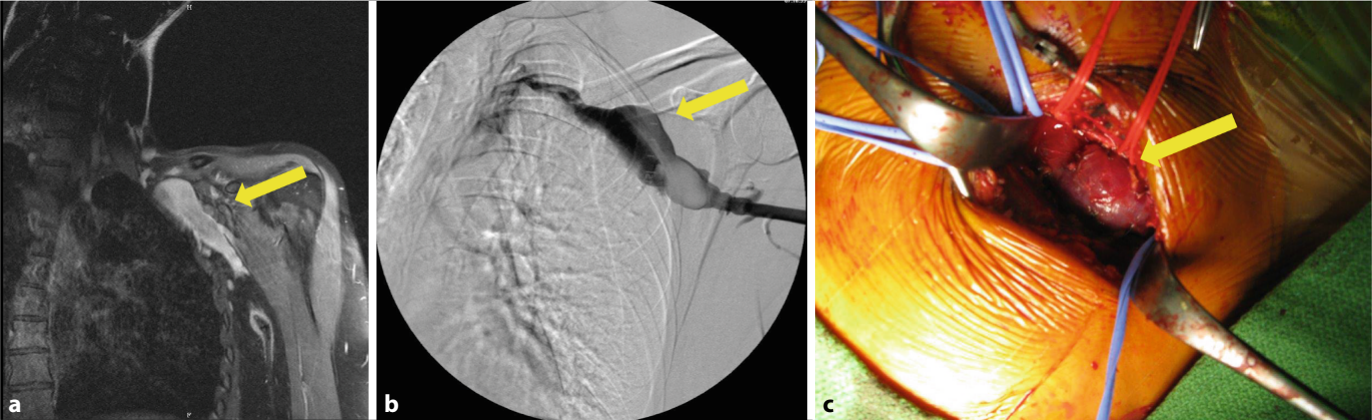
Fall 5: Venöses Aneurysma der V. axillaris links. **a** MR-Angiographie in koronarer Schichtung mit Darstellung des venösen Aneurysmas (*gelber Pfeil*), **b** Venöse Angiographie der V. axillaris links (*gelber Pfeil*), **c** Intraoperativer Befund des venösen Aneurysmas der V. axillaris links (*gelber Pfeil*)

Schmerzen und Druckgefühl im Bereich der linken Schulter seit ca. 5 Jahren führten bei einer 39-jährigen Patientin zur weiterführenden Diagnostik mittels MRT. Hier stellte sich eine Raumforderung im Bereich des Plexus brachialis (Abb. 5a) links dar, die sich in der Phlebographie (Abb. 5b) zweifelsfrei als polylobulierte aneurysmatische Läsion der V. axillaris proximal der Mündungsstelle der V. cephalica mit einem Durchmesser von 36 × 90 mm darstellte. Die operative Versorgung erfolgte durch eine tangentiale Abtragung der Vorderwand des Aneurysmas und Übernähung der Vorderwand mittels 5 × 0-Prolenenaht in fortlaufender Nahttechnik auf eine Länge von ca. 5 cm (Abb. 5c). Nach perioperativem Bridging mit Enoxaparin (Clexane®, Sanofi-Aventis, Frankfurt, Deutschland) in gewichtsadaptierter Dosierung wurde die Patientin postoperativ auf Falithrom eingestellt.

#### Fallbericht 6

**Abb. 6:**
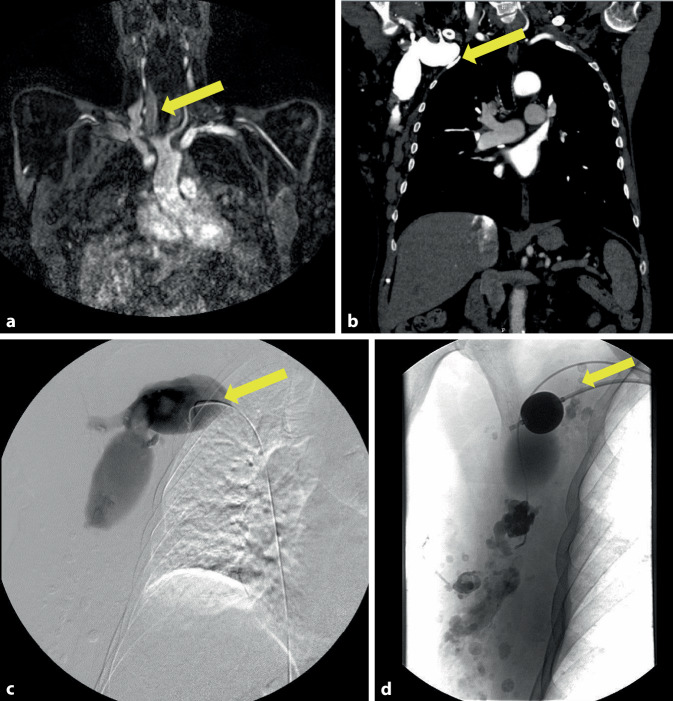
Fall 6: Aneurysmatische venöse Malformation mit Abfluss in eine ektatische V. subclavia rechts sowie ein venöser Abfluss über die V. azygos. **a** MR-Angiographie in koronarer Schichtung mit Darstellung des venösen Aneurysmas (*gelber Pfeil*), **b** CT-Angiographie des venösen Aneurysmakomplexes in koronarer Darstellung (*gelber Pfeil*), **c** Angiographische Darstellung des venösen Aneurysmas bei der Embolisation mit Embolisationskatheter (*gelber Pfeil*), **d** Embolisiertes venöses Aneurysma mit Blockungsballon (*gelber Pfeil*)

Der 66-jährige Patient wurde aufgrund rezidivierender Lungenembolien in einem Krankenhaus der Grund- und Regelversorgung aufgenommen und konservativ therapiert. Im Zuge der subtilen weiterführenden Diagnostik der vorliegenden Gefäßverhältnisse schlossen sich MRT (Abb. 6a) sowie Kontrastmittel-CT des Thorax (Abb. 6b) an. Dabei konnte eine aneurysmatische venöse Malformation mit Abfluss in eine ektatische V. subclavia rechts sowie ein venöser Abfluss über die V. azygos unter Hinweis auf eine frische Lungenarterienembolie im Bereich der rechten Unterlappenarterie bestätigt werden. Die Versorgung der venösen aneurysmatischen Läsion erfolgte im Rahmen eines Stufenkonzeptes zunächst mittels erster Embolisation (Abb. 6c), am Folgetag mit einer zweiten Embolisation und Blockung des Aneurysmahalses (Abb. 6d), anschließender operativer Ligatur des Aneurysmahalses unter Entfernung des Blockungsballons. Bis auf ein subkutanes Hämatom waren postoperativ keine Komplikationen im Verlauf zu verzeichnen, sodass der Patient nach erneuter Falithromeinstellung in die ambulante Betreuung entlassen werden konnte. Eine Kontroll-CT‑A nach 8 Monaten zeigte eine vollständige Embolisation des venösen Aneurysmas und der aneurysmatischen Aussackung der V. subclavia sowie Ektasie im Bereich der V. azygos.

### Abdominelle venöse Aneurysmen

Retroperitoneale venöse Aneurysmen, die von der unteren Hohlvene, den Beckenvenen oder den Nierenvenen ausgehen, sind ebenfalls äußerst selten. Die Diagnostik kann in solchen Fällen schwierig sein, da sie oft Weichteiltumoren nachahmen [26]. Abbot et al. [1] klassifizierten diese Aneurysmen je nach Ursache als primär oder sekundär. Sekundäre venöse Aneurysmen werden als Folge von AV-Fisteln, proximaler Obstruktion oder kardiovaskulären Anomalien angesehen, die den Fluss oder Druck im Venensystem erhöhen. Obwohl die wahre Ätiologie nicht vollständig verstanden ist, werden andere Fälle, die ohne Hinweise auf solche Ursachen auftreten, als primäre Aneurysmen betrachtet [28]. Angesichts eines potenziellen Risikos für thromboembolische Komplikationen – Symptome, die sich aus der Kompression benachbarter Strukturen oder sogar einer Ruptur ergeben – ist nach der Diagnose die elektive Behandlung von Beckenvenenaneurysmen angezeigt. Es gibt keine standardisierte Behandlung, sodass in der Literatur über die tangentiale Aneurysmektomie mit lateraler Venorrhaphie, Aneurysmaresektion mit einer Patchplastik, Staplernaht oder Polytetrafluorethylen(PTFE)-Transplantate berichtet wurde [28].

#### Fallbericht 7

**Abb. 7:**
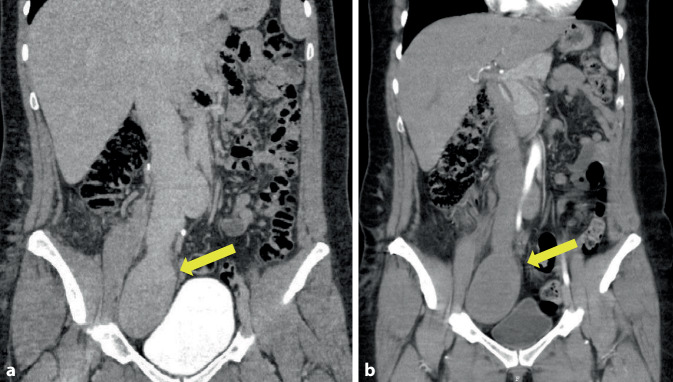
Fall 7: Aneurysma der rechten Beckenvene und V. cava inferior. **a** CT-Angiographie in koronarer Schichtung bei Erstdiagnose des venösen Aneurysmas der V. iliaca und V. cava inferior (*gelber Pfeil*), **b** CT-Angiographie-Verlaufskontrolle (koronar) nach 10 Jahren (*gelber Pfeil*)

Bei einer 34-jährigen Patientin war im Rahmen einer gynäkologischen Routineuntersuchung eine aneurysmatische Aufweitung der rechten Beckenvene auf 7 cm im Querdurchmesser aufgefallen. In der sich anschließenden CT-Angiographie konnte neben der Bestätigung des Befundes der rechten Beckenachse ebenfalls eine Ektasie der V. cava inferior nachgewiesen werden (Abb. 7a). Weder klinisch noch diagnostisch ließen sich Zeichen einer Thrombose oder Embolie nachweisen. Der Patientin wurde anschließend die operative Versorgung zur Protektion von Thrombenbildung und Ruptur vorgeschlagen. In einer auswärtigen Zweitbeurteilung wurde der Patientin bei bisher unauffälligem klinischem Verlauf nur eine Therapie in Risikosituationen mittels Thromboseprophylaxe durch Heparingabe und Tragen von Oberschenkelkompressionsstrümpfen empfohlen. Im Verlauf einer Routinekontrolle nach 10 Jahren zeigte sich in der radiologischen Diagnostik (Abb. 7b) zwar ein konstanter Befund, jedoch sprachen erhöhte D‑Dimere im Labor für eine aktivierte Gerinnung, sodass die Patientin nach einer Diagnostik zum Ausschluss von Lungenembolie und Thrombophilie auf ein Phenprocoumonpräparat zur Prophylaxe eingestellt wurde. Hierunter entwickelte die Patientin eine Spontanblutung im Urogenitaltrakt, die urologisch behandelt werden musste. Im Zuge der urologischen Behandlung durch Zystoskopie und Harnleiterschienung, flankiert von einer Vitamin-K-Gabe und supportiven Behandlung mit niedermolekularem Heparin in therapeutischer Dosis, erlitt die Patientin eine tödliche Lungenembolie.

### Eigene Patientenklientel

Zwischen 2005 und 2023 wurden insgesamt 11 Fälle venöser Aneurysmen bei Patienten im Alter von 30 bis 84 (Mittelwert: 52,5, Median: 50) Jahren eruiert, wobei bei einem Patienten nach 2 Jahren ein Rezidiv operiert werden musste. Das Geschlechterverhältnis betrug 7:3 (7 Frauen, 3 Männer). Von der anatomischen Region war die V. poplitea mit 36,4 % (*n* = 4) am häufigsten betroffen, gefolgt von der V. jugularis interna und V. axillaris/subclavia mit 18,2 % (*n* = 2). Die V. cava inferior, die V. iliaca communis und die V. cubiti media traten nur je einmal auf (9,1 %). Beim Nebenerkrankungsprofil ergab sich keine gehäufte Auffälligkeit. So waren die bei arteriellen Aneurysmen bekannten Risikofaktoren wie Hypertonus und koronare Herzkrankheit (KHK) nur bei einer Patientin zu eruieren, die zudem noch an einem Pseudoxanthoma elasticum (Groenblad-Strandberg Syndrom), einer autosomal-rezessiv vererbten Multisystemerkrankung, litt. Eine COPD oder Krebserkrankung fand sich jeweils nur bei einem Patienten, ein Vorhofflimmern bei zwei Patienten. Das Durchschnittsgewicht aller Patienten betrug 76,2 kg, die durchschnittliche Körpergröße 171,8 cm. In 9 Fällen erfolgte eine operative Versorgung der Aneurysmen. Dabei kamen eine

- tangentiale Resektion der Aneurysmawand und fortlaufende Raffungsnaht (*n* = 5),
- Resektion des Aneurysmas und Interposition einer 8-mm-GORE-TEX®Vascular-Graft-Prothese (*n* = 1),
- Ligatur des Aneurysmas (*n* = 2) und
- Ligatur mit anschließender Resektion des Aneurysmas (*n* = 1)

zur operativ-technischen Anwendung. Die durchschnittliche Operationszeit betrug 76 min. Dabei musste in keinem Falle peri- und postoperativ eine Bluttransfusion erfolgen. Bis auf die einfache Ligatur des Aneurysmas der V. cubiti media erfolgte unmittelbar postoperativ in allen operativen Fällen standardisiert die intravenöse Gabe von 500 IE Heparin/h und Erhöhung der Heparindosis am 1. postoperativen Tag mit einer Ziel-PTT von 50–60 s. Beim Ausbleiben von Blutungsstigmata und klinisch stabilem Zustand des Patienten wurde in 6 Fällen vor Einstellung einer Antikoagulation auf Enoxaparin (Clexane®) in gewichtsadaptierter Dosis subkutan umgestellt. In 2 Fällen erfolgte die sofortige Umstellung auf ein direktes orales Antikoagulans. Vor Entlassung aus der stationären Behandlung wurde in 8 Fällen auf eine orale Antikoagulation eingestellt, wobei sich ab 2017 die Einstellung auf ein direktes orales Antikoagulans (DOAK; Falithrom: *n* = 4, DOAK: *n* = 4) durchsetzte. Bis auf ein leichtes subkutanes Hämatom und ein Serom im Wundbereich in jeweils einem Fall waren keine weiteren Wundkomplikationen aufgetreten. Das histologische Untersuchungsergebnis des Aneurysmaresektates beschrieb in 4 Fällen eine myxoide Degeneration/Auflockerung, atypische Zellen wurden in keinem Fall beschrieben. Nur bei 5 operierten Patienten wurde ein follow-up nach 1 Jahr dokumentiert, wobei in 3 Fällen sich für eine Fortführung der Antikoagulation mit einem DOAK und in einem Fall mit einem Vitamin-K-Antagonisten ausgesprochen wurde.

## Diskussion

Da venöse Aneurysmen seltene Entitäten sind, ist die Implementierung einer validen Behandlungsstrategie anhand der Einzelfallbeschreibungen oder Fallsammlungen schwierig. Daher soll die Beurteilung der eigenen Behandlungsergebnisse mit der aktuellen Literatur verglichen und allgemeingültige Behandlungsempfehlungen erstellt werden. Die Entstehung venöser Aneurysmen ist anhaltend ungeklärt und stützt sich auf verschiedene Modelle. Eine Untersuchung der Metalloproteinaseexpression in venösen Aneurysmen von Irwin et al. [8] zeigte neben strukturellen Veränderungen in der Venenwand eine erhöhte Expression von Metallproteinasen MMP‑2, MMP‑9 und MMP-13 im Vergleich zur „normalen“ Stammvene und zur varikösen Vene, was auf eine mögliche kausale Rolle dieser MMPs in ihrer Pathogenese hindeutet. Die populärste Theorie für die Entstehung venöser Aneurysmen ist ein fokaler Verlust der normalen Bindegewebskomponenten der Venenwand aufgrund einer angeborenen Unterentwicklung oder einem degenerativen Verlust mit zunehmendem Alter, was zu Wandschwäche und Anfälligkeit für eine Dilatation führt [25]. Die von Lev und Saphir [9] beschriebene Endophlebohypertrophie und Endophlebosklerose sind histologische Manifestationen dieser Prozesse. In den eigenen histologischen Untersuchungspräparaten zeigte sich überwiegend eine myxoide Degeneration, was der o. g. Theorie entspricht. Entzündliche Veränderungen oder Atypien wurden nicht nachgewiesen. Ebenfalls auffällig in der eigenen Patientenklientel war das Überwiegen des weiblichen Geschlechts. Da das weibliche Geschlecht als ein unabhängiger Risikofaktor für eine primäre Varikosis gilt [18], ist eine Disposition in Bezug auf venöse Aneurysmen zu vermuten. Die weibliche Dominanz bei venösen Poplitealaneurysmen wurde ebenfalls in einem systematischen Review von Bergquist et al. [3] bestätigt. Diese Arbeitsgruppe bestätigte auch das mittlere Alter von Anfang 50, was in der hiesigen Kohorte bei 52,5 Jahren lag bei einer Altersspanne von 30 bis 84 Jahren. Bei der Lokalisation der venösen Aneurysmen sind die unteren Extremitäten am häufigsten betroffen, insbesondere die V. poplitea, gefolgt von Aneurysmen des Kopfes und Halses, der Bauchvenen und der Thoraxvenen [22]. Dieser Fakt bestätigte sich auch bei den eigenen ausgewerteten Fällen. Die Ätiologie der Poplitealvenenaneurysmen ist unklar, kann aber angeborene Gefäßfehlbildungen, Traumata, Entzündungen oder lokalisierte degenerative Veränderungen umfassen [30]. Vielleicht ist die Poplitealregion als intensiv genutztes Bewegungssegment des Menschen einem besonderen Scheerstress der doch zarteren Venenwand ausgesetzt, sodass die venösen Aneurysmen hier am häufigsten vorkommen. In den eigenen behandelten Fällen konnten wir keine Risikofaktoren differenzieren, wobei die Zahl der Patienten für eine statistisch valide Aussage zu gering ist. Ein Zusammenhang zwischen dem beschriebenen Aneurysma der V. jugularis interna und der autosomal-rezessiv vererbten Multisystemerkrankung Pseudoxanthoma elasticum (Groenblad-Strandberg-Syndrom) ist bisher in der Literatur nicht beschrieben worden, sodass sicherlich auch hier kein pathogenetischer Zusammenhang zu sehen ist. Auch die konstitutionellen Basisdaten der Patienten ergeben in der eigenen Patientengruppe mit dem Durchschnittsgewicht von 76,2 kg und einer durchschnittlichen Körpergröße von 171,8 cm keinen richtungsweisenden Parameter, um aneurysmagefährdete Patienten zu identifizieren.

Das weitestgehend standardisierte perioperative Management mit low-dose-Heparinisierung in der unmittelbaren postoperativen Phase, Bridging auf ein niedermolekulares Heparin in der ersten Wundheilungsphase und Ein-(oder Wiederein-)stellung auf ein orales Antikoagulans hat sich in der eigenen Klinik bewährt. Es waren bis auf ein subkutanes Hämatom und ein Serom keine perioperativen Blutungen oder Gaben von Erythrozytenkonzentraten notwendig. Ebenfalls konnten keine Thrombosen oder perioperativen Embolien verzeichnet werden. Die durchgeführten chirurgischen Operationsmethoden finden sich ebenfalls im Einklang mit der aktuellen Literatur. Zu den chirurgischen Behandlungsoptionen für venöse Aneurysmen gehören Ligatur, Resektion mit End-zu-End-Anastomose, Resektion mit Interpositionstransplantat und tangentiale Aneurysmektomie mit lateraler Venorrhaphie [23]. Die häufigste Methode in der eigenen Klinik war die tangentiale Resektion der Aneurysmawand und fortlaufende Raffungsnaht, gefolgt von der Ligatur des Aneurysmas, von der Resektion des Aneurysmas und Interposition einer 8‑mm-GORE-TEX®Vascular-Graft-Prothese sowie einer Ligatur mit anschließender Resektion des Aneurysmas. Bei einem Patienten kam es nach Aneurysmaresektion und Raffungsnaht zu einem Rezidiv. Hier zeigt sich die Notwendigkeit einer postoperativen Verlaufskontrolle, die leider in der eigenen Patientenklientel unvollständig verblieb. Es erfolgte hier der Methodenwechsel auf die Resektion des Aneurysmas und Interposition einer PTFE-Prothese.

Viel diskutiert ist die postoperative Notwendigkeit und Dauer einer Antikoagulation. Aus dem eigenen Erfahrungsschatz scheint die Antikoagulation prä- und postoperativ, insbesondere bei den venösen Aneurysmen der Extremitäten, Beckenvenen und der V. cava inferior, geboten zu sein. Anhand der eigenen Statistik sieht man den Wechsel des Einsatzes der Vitamin-K-Antagonisten auf die DOAKs ab Mitte der 2010er-Jahre. Wie auch bei der Behandlung von Lungenembolien und Thrombosen scheint der Einsatz von DOAKs in der praktischen Handhabung und klinischen Wirksamkeit die Vitamin-K-Antagonisten verdrängt zu haben. Über die Länge der Antikoagulation postoperativ herrscht weiterhin Uneinigkeit, wobei die meisten Autoren diese für 3 bis 6 Monate empfehlen [6, 17, 23]. Dies bestätigt sich auch in der eigenen postoperativen Handhabung.

Da bezüglich der postoperativen Antikoagulation keine evidenzbasierten Daten vorliegen, ist sicherlich bezüglich der Indikation zur Antikoagulation, der Antikoagulationsform und der Dauer eine patientenindividualisierte Entscheidung zu treffen. Dabei sollten Aspekte der gefäßchirurgischen Rekonstruktionsform, der verwendeten Materialien sowie der Lokalisation des venösen Aneurysmas mit in die Betrachtung einbezogen werden. In der retrospektiven Studie von Noppeney *et al*. [15] aus dem Jahr 2019, in der insgesamt 39 Patienten mit venösen Poplitealaneurysmen eingeschlossen wurden, erfolgte im überwiegenden Teil die tangentiale Resektion und Direktnaht. In dieser Studie erhielten die Patienten eine 3‑monatige therapeutische Antikoagulation unter der Annahme, dass dies dem Endothel genügend Zeit ließe, die Nahtlinie in der Vene zu überwachsen. Eine systematische Analyse venöser poplitealer Aneurysmen durch Fernandez *et al*. kam zu dem Ergebnis, dass 12 Autoren der analysierten Publikationen eine 3‑monatige Antikoagulation durchführten und 11 Autoren eine 6‑monatige oder dauerhafte Antikoagulation anwendeten [12]. Der überwiegende Anteil an Publikationen zu Beckenvenenaneurysmen gibt keine Empfehlung bzw. Erläuterung zur Entscheidungsfindung zur Antikoagulation. Nach Ansicht der Autoren handelt es sich um monströse Aneurysmen, die nach meist genutzter tangentialer Resektion eine große thrombogene Nahtfläche besitzen und daher postoperativ mindestens ein halbes Jahr antikoaguliert werden sollten. Nachfolgend ist am ehesten eine individualisierte Entscheidung in Abhängigkeit von der Verlaufskontrolle zu empfehlen, da Rezidive und Thrombosen beschrieben wurden. Des Weiteren sollte die Länge der postoperativen Antikoagulationsphase von präoperativ aufgetretenen embolischen Ereignissen und von der Komplexität des Eingriffs abhängig gemacht werden. Sessa *et al*. empfehlen den Patienten Kompressionsstrümpfe und niedermolekulares Heparin für 3 Wochen nach tangentialer Aneurysmektomie und bei o. g. Risikofaktoren eine orale Antikoagulationstherapie für 3 Monate [20].

In der gesichteten Literatur zur chirurgischen Versorgung venöser Aneurysmen fanden sich keine Hinweise, dass die Antikoagulationsdauer von Operationsverfahren mit alloplastischem Material abhängig gemacht wurde. In Anlehnung an die Publikation von Gloviczki *et al*. [7], die nach Rekonstruktionen nichtmaligner Verschlussprozesse großer Venen über Erfahrungen mit ePTFE berichteten und die lebenslange Antikoagulation favorisierten, könnte die Materialwahl in die Entscheidungsfindung mit einbezogen werden.

Komplikationen von Beckenvenenaneurysmen sind hauptsächlich embolische Ereignisse oder Rupturen [10], sodass in dem geschilderten Fall der 34-jährigen Patientin durchaus eine Indikation zur operativen Versorgung zu sehen ist. Gerade auch im Hinblick auf das jugendliche Alter und das weibliche Geschlecht erscheint das Rupturrisiko eines solchen monströsen Aneurysmas beispielsweise bei einer Schwangerschaft mit nachfolgendem Partus deutlich erhöht. Eine Einbeziehung der V. cava inferior wäre in Abhängigkeit vom intraoperativen Lokalbefund zur Konditionierung des venösen Rückflusses überlegenswert gewesen. Die intraoperative Anlage einer AV-Fistel zur Flussbeschleunigung in der Beckenachse erscheint aus Sicht der Autoren nicht empfehlenswert, da bei der großen Resektionsfläche von einer erhöhten Nachblutungsgefahr bei begleitender perioperativer Heparinisierung ausgegangen werden muss. Empfohlen wird eine konsequente Kompressionstherapie oder intermittierende pneumatische Kompression [21].

### Stärken

Die hier vorgestellten Fallbeispiele waren durch das Bildmaterial gut darzustellen und zu illustrieren. Die aus der konstanten Handhabung der seltenen Entitäten entstandenen Behandlungsergebnisse bestätigen das operative und perioperative Management. Aufgezeigt werden konnte ebenfalls der Vorteil eines interdisziplinären individuellen Fallmanagements durch Kombination interventionell-radiologischer und offen-gefäßchirurgischer Behandlungsverfahren.

### Limitationen

Die retrospektive Betrachtungsweise der eher begrenzten Anzahl der eruierten Fälle schränkt eine verallgemeinernde Aussagekraft des gesammelten Datenmaterials ein. Auch durch das zu selten adäquat erfolgte, aber zu favorisierende follow-up lassen sich insbesondere hinsichtlich der interessanten Fragen wie nach der Notwendigkeit und Länge der postoperativen Antikoagulation nicht näher bestimmen und keine konkreteren Aussagen oder gar allgemeingültig abzuleitende Empfehlungen treffen.

### Ausblick

Eine zentrale (ggf. bundesweite) Datenbank, in der solche seltenen Fälle, wie beschrieben, registriert und aufgearbeitet werden, wäre für die wissenschaftliche Erstellung von Behandlungsstrategien sicherlich wertvoll.

## Fazit für die Praxis

Die Seltenheit des Auftretens der venösen Aneurysmen sollte Anlass geben, diese Fälle zentral zu sammeln und auszuwerten. Die chirurgische Behandlung ist meist unproblematisch und mit eher wenigen Komplikationen behaftet. Das Risiko von Lungenembolien scheint bei venösen Aneurysmen der Extremitäten, Beckensegmente und V. cava inferior deutlich erhöht zu sein, während venöse Aneurysmen im Kopf- und Halsbereich deutlich weniger dazu neigen. Die peri- und postoperative Antikoagulation hat sich an die Entwicklung der Antikoagulanzien angepasst, weshalb die Falithromtherapie zugunsten der Therapie mit DOAKs weitestgehend umgestellt wurde. Eine unmittelbar postoperative low-dose-Heparinisierung mit anschließendem therapeutischen Bridging mit einem niedermolekularen Heparin vor Einstellung auf ein Antikoagulans scheint die perioperative Phase bezüglich operationsbedingter Komplikationen nach eigener Erfahrung abzusichern.

## Data Availability

Die erhobenen Datensätze können auf begründete Anfrage in anonymisierter Form beim korrespondierenden Autor angefordert werden. Die Daten befinden sich auf einem Datenspeicher im Arbeitsbereich Gefäßchirurgie, Klinik für Allgemein‑, Viszeral‑, Gefäß- und Transplantationschirurgie, Uniklinikum Magdeburg A. ö. R.

## Footnotes

1 Aus Gründen der besseren Lesbarkeit wurde nur ein Geschlecht in den Formulierungen verwendet, wobei stets alle Formen in der Betrachtung einbezogen sind.
